# IL-33 Alarmin and Its Active Proinflammatory Fragments Are Released in Small Intestine in Celiac Disease

**DOI:** 10.3389/fimmu.2020.581445

**Published:** 2020-10-08

**Authors:** Federico Perez, Carolina N. Ruera, Emanuel Miculan, Paula Carasi, Karen Dubois-Camacho, Laura Garbi, Luciana Guzman, Marcela A. Hermoso, Fernando G. Chirdo

**Affiliations:** ^1^ Instituto de Estudios Inmunológicos y Fisiopatológicos (IIFP), UNLP, CONICET, CIC PBA, Departamento de Ciencias Biológicas, Facultad de Ciencias Exactas, Universidad Nacional de La Plata, La Plata, Argentina; ^2^ Innate Immunity Laboratory, Immunology Program, Faculty of Medicine, Biomedical Sciences Institute, Universidad de Chile, Santiago, Chile; ^3^ Servicio de Gastroenterologia, Hospital General San Martin, La Plata, Argentina; ^4^ Servicio de Gastroenterologia, Sor Maria Ludovica, Hospital de Niños, La Plata, Argentina

**Keywords:** IL-33, ST2, celiac disease, alarmins, inflammation, innate immunity, small intestine, gut immunity

## Abstract

Initially described as Th2 promoter cytokine, more recently, IL-33 has been recognized as an alarmin, mainly in epithelial and endothelial cells. While localized in the nucleus acting as a gene regulator, it can be also released after injury, stress or inflammatory cell death. As proinflammatory signal, IL-33 binds to the surface receptor ST2, which enhances mast cell, Th2, regulatory T cell, and innate lymphoid cell type 2 functions. Besides these Th2 roles, free IL-33 can activate CD8^+^ T cells during ongoing Th1 immune responses to potentiate its cytotoxic function. Celiac Disease (CD) is a chronic inflammatory disorder characterized by a predominant Th1 response leading to multiple pathways of mucosal damage in the proximal small intestine. By immunofluorescence and western blot analysis of duodenal tissues, we found an increased expression of IL-33 in duodenal mucosa of active CD (ACD) patients. Particularly, locally digested IL-33 releases active 18/21kDa fragments which can contribute to expand the proinflammatory signal. Endothelial (CD31^+^) and mesenchymal, myofibroblast and pericyte cells from microvascular structures in villi and crypts, showed IL-33 nuclear location; while B cells (CD20^+^) showed a strong cytoplasmic staining. Both ST2 forms, ST2L and sST2, were also upregulated in duodenal mucosa of CD patients. This was accompanied by increased number of CD8^+^ST2^+^ T cells and the expression of T-bet in some ST2^+^ intraepithelial lymphocytes and *lamina propria* cells. IL-33 and sST2 mRNA levels correlated with IRF1, an IFN induced factor relevant in responses to viral infections and interferon mediated proinflammatory responses highly represented in duodenal tissues in ACD. These findings highlight the potential contribution of IL-33 and its fragments to exacerbate the proinflammatory circuit and potentiate the cytotoxic activity of CD8^+^ T cells in CD pathology.

## Introduction

Interleukin-33 (IL-33) is a 30 kDa protein belonging to the IL-1 family, that is mainly expressed in the nucleus of fibroblasts, subepithelial myofibroblasts (SEMFs), keratinocytes, adipocytes, endothelial and epithelial cells from different tissues (1). Inside the nucleus, IL-33 works as a nuclear factor influencing the expression of several proinflammatory genes, such as *IL8* and *IL6* (2). Interestingly, like other IL-1 family members such as IL-1α, IL-33 acts as an alarmin, being released from cells during a programmed cell death like necroptosis and pyroptosis, stimulating different immune responses (2). In contrast, when cells die by apoptosis, IL-33 is cleaved by active caspase 3 or 7, producing a non-bioactive form.

Remarkably, enzymes secreted by neutrophils or mast cells like cathepsin G, neutrophil elastase, tryptase or chymase may cleave free IL-33 producing diverse mature fragments of 18 to 21 kDa, having stronger bioactivities than the full-length form (3, 4).

Soluble IL-33 was initially linked to a strong capacity to stimulate Type 2 immune responses, through its actions on Th2, Mast cells and ILC2 cell (5–7). However, increasing evidence demonstrated that IL-33 is also able to promote Type-1 immune responses (8), CD8^+^ T cytotoxic cells (9) and invariant NKT cells activation (10), as well as, support regulatory T (Treg) cell responses (11).

The cellular IL-33 receptor complex “IL-33R” is made up of 2 transmembrane proteins known as ST2L which, together with the accessory protein IL-33Rβ, binds to IL-33 mediating intracellular signaling through the cytosolic adaptor MyD88, which in turn activates the MAPK and other kinase cascades, such as NF-kB, JNK, p38, and PI3K (12). ST2L is predominantly expressed by Th2 lymphocytes, ILC2, Tregs, CD8^+^ T lymphocytes, some active Th1 lymphocytes, monocytes, mast cells, NKT and NK cells, macrophages, eosinophils and mesenchymal cells (5, 13, 14). IL-33 also has a soluble binding protein, named sST2, which is a spliced variant of the *IL1RL1* gene lacking the transmembrane and intracellular domain of the ST2L. When sST2 binds to extracellular IL-33, it avoids the cellular signaling through the ST2L, therefore sST2 acts as a decoy receptor regulating this axis (2).

IL-33 can be an important factor on gut homoeostasis promoting a regulatory environment through its direct action on Treg and ILC2 populations (15). Macrophages, ILC2 and Treg cells can participate in the healing process dampening an excessive local inflammatory response (16, 17). Moreover, IL-33 is involved in protection of gut epithelial barrier by regulation of IgA production and secretion of RegIIIγ (18, 19). Furthermore, IL-33 allows differentiation of myofibroblast, which makes it an important factor in wound healing, and fibrosis processes (20, 21). In the last years, IL-33 has become a novel focus of attention in inflammatory disorders, such as inflammatory bowel disease (22), infectious disease (23), and autoimmunity (24).

Since effects of IL-33 vary, being pro-tolerogenic or proinflammatory depending on the context of the immune response (13), to understand its role it is necessary to consider which cells are involved as well as, the microenvironment where IL-33 was released. Though the role of IL-33 has been evaluated in gut inflammation (inflammatory bowel disease) (22), its function has not been fully described in human small intestine. Particularly, its capacity to act as an alarmin or cytokine, led us to explore its role in the celiac disease (CD).

CD is a chronic gastrointestinal disorder developed in genetically predisposed individuals after gluten intake. Typical changes in proximal small intestine include villus atrophy, crypt hyperplasia and lymphocytic infiltration in both epithelium and *lamina propria*. The mucosal damage is driven by gluten-specific CD4^+^ Th1 lymphocytes, that recognize gluten-derived peptides presented in HLA-DQ2 or DQ8 molecules. However, innate mechanisms must also be involved (25). IL-33 and sST2 levels are increased in sera, as well their expression in duodenum of pediatric CD patients, unveiling a link between IL-33/ST2 axis and CD pathogenesis (26). In this work, we aimed to identify the cells expressing molecules associated to IL-33/ST2 axis, to assess the participation of IL-33 alarmin in potentiating local inflammation and evaluate the connection with inflammatory pathways in untreated celiac disease conditions.

## Materials and Methods

### Patients

Duodenal biopsies and blood samples were collected from active celiac disease patients (ACD) and non-celiac (NC) individuals during the routine protocol for CD diagnosis in the Gastroenterology Units of Hospital Sor María Ludovica (pediatric patients) and Hospital San Martin (adult patients). ACD was diagnosed by histological examination of duodenal biopsies, serology (anti-transglutaminase 2, anti-deamidated gliadin peptides and anti-endomysium antibodies), and the evaluation of clinical presentation. The relevant clinical data of each patient is presented in **Supplementary Table 1**.

### Ethics Statement

All participants provided informed consent. The study was approved by the Ethical Committees of both Public Health Institutions and performed according to human experimental guidelines. Clinical investigation was conducted according to Declaration of Helsinki principles with participants identified only by number.

### Levels of ST2 and IL-33 in Serum

The concentration of IL-33 and sST2 in serum samples (100 μl) was determined by commercial ELISA kits according to the manufacturer’s instructions (DuoSet, R&D Systems, Minneapolis, MN, USA). Each sample was measured in duplicated and average results were expressed as pg/mL.

### Immunofluorescent Microscopy

Immunofluorescent assays for IL-33 were performed using paraffine embedded samples. Duodenal samples were fixed with 4% w/v formalin for 24 h at room temperature. Samples were dehydrated and included in paraffin for further sectioning. Sections of 10 μm of duodenal paraffin-embedded tissues were deparaffinized and rehydrated in distilled water. Antigen retrieval was performed by heat treatment in a Commercial buffer CITRA PLUS Solution (Cat. HK080-9K, Biogenex). The immunofluorescence analysis for ST2 was performed using formalin fixed frozen samples. The tissues were fixed with 4% w/v formalin, 30% w/v sucrose in phosphate buffer for about 24 h at 4°C. After that, we included the tissues in a polyvinyl matrix on a liquid nitrogen bath. Sections of 10 μm of samples where used for immunofluorescence analysis. The tissues samples were blocked using horse serum or fetal bovine serum at 5% in PBS, and then incubated with primary antibodies for: CD90 (Abcam, Cat# ab133350, RRID: AB_11155503, USA), CD31 (Abcam, Cat# ab187377, RRID: AB_2756834, USA), CD11c (Abcam, Cat# ab52632, RRID: AB_2129793, USA), CD45 (Abcam, Cat# ab30470, RRID: AB_726544, USA); CD138 (DAKO, Cat# M7228, RRID: AB_2254116, USA), CD3 (DAKO, Cat# M7254, RRID: AB_2631163, USA); CD64 (Biolegend, Cat# 305012, RRID: AB_528867, USA), CD20 (Affimetrix, Cat# 17-0209-71, RRID: AB_469361, USA); T-Bet (Affimetrix, Cat# 45-5825-80, RRID: AB_953658, USA); for human IL-33 (R&D Systems, Cat# AF3625, RRID: AB_1151900, USA) and ST2 (R&D Systems, Cat# AF523, RRID: AB_2125427, USA); mouse IL-33 (R&D Systems, Cat# AF3626, RRID: AB_884269, USA) and suitable isotypes controls from the same brands. After an overnight incubation, the samples were washed with PBS buffer and then incubated with secondary antibodies: anti-IgG(Mouse)-Alexa647 (Abcam, Cat# ab150107, USA), anti-IgG(Rabbit)-Alexa647 (Abcam, Cat# ab150075, RRID: AB_2752244, USA), anti-IgG(Goat)-Alexa488 (ThermoFisher, Cat# A32814, RRID: AB_2762838, USA). For nuclear staining we used DAPI (ThermoFisher, Cat# D3571, RRID: AB_2307445, USA) at 1 μg/ml.

### Images Analysis

Images were obtained using a SP5 Leica confocal microscope or Apotome Zeiss microscope. The Fluorescence staining intensity was obtained under the same acquisition parameters (light intensity, sensor gain, and exposure time). The images were analyzed by the FIJI software. Importantly, we performed a manual segmentation of *lamina propria* sections as well as epithelium compartment to quantify Total Fluorescent Intensity per tissue area. We also determined a threshold value of the fluorescent signals to be able to quantify it on segmented areas. The threshold levels for quantification were defined as grater as mean of isotype control plus 2 standards deviations. Total Fluorescence per area was defined as relative units of Fluorescence Intensity/Area (μm^2^), named here as “Relative Fluorescence Density” or RFD. The total area inside of the segmented compartments was calculated with a threshold method known as “Huang” (27). This strategy allows the use of the unspecific staining of cells as an indicative of the total area, avoiding counting area of empty space of the tissue or unprecise manual segmentation. On the other hand, cell proportion was manually calculated counting positive cells inside a manually predefined area on each histological compartment.

We performed an increment of contrast and brightness in all the pixels for the images presented in this study in order to make the staining clearer and more distinguishable for the readers. However, these modifications were not performed during the image’s analysis by the RDF or manual cell counting.

### Cell Culture

HT-29 cells were cultured in Dulbecco’s Modified Eagle Medium (DMEM) (Gibco, Invitrogen, USA) supplemented with 10% v/v inactivated fetal calf serum (Gibco, Invitrogen, USA) with streptomycin at 100 μg/ml and penicillin G at 100 U/ml. Cells were cultured in 24 wells plate for 6, 24 or 48 h after incubation with 100 μg/mL p31-43 (Genecust, Luxenburgh), 24 h with 50 ng/mL IFN*γ* (R&D systems, Cat #285-IF-100, USA) and 6 h with 10 μg/mL Poly I:C (Sigma Aldrich, Cat # P1530-100MG, USA).

### Western Blot Analysis

Duodenal biopsies were frozen on dry ice immediately after obtention. Total protein was determined by standard procedures. Briefly, tissue samples were incubated with protease inhibitor O complete (Roche, USA), NP-40 (1% v/v) in a Tris buffer (pH:8.4). The samples were minced using mechanical disruption. Total protein concentration was determined following the instructions of producer (Pierce™ BCA Protein Assay Kit, Thermofisher, USA). Thirty micrograms of whole protein fraction were added per well on a 10% or 14% acrylamide/bisacrylamide gel for ST2 or IL-33, respectively. The separation was performed with a mini-Protean II Hercules (Bio-Rad, USA). Then, protein bands were transferred to a nitrocellulose blotting membrane (Amersham™ Protran^®^ Western blotting membranes, USA). After the corresponding blocking step, primary antibodies were incubated on blocking buffer for 16 h at 4°C (5% w/v skimmed milk on 0.1% Tween20-Tris Buffer). Primary antibodies used: anti-mouse IL-33 (R&D Systems, Cat# AF3626, RRID: AB_884269, USA) at 5 μg/ml, anti-human IL-33 (R&D Systems, Cat# AF3625, RRID: AB_1151900, USA) at 5 μg/ml, anti-human ST2 (R&D Systems, Cat# AF523, RRID: AB_2125427, USA) at 1 μg/ml, the loading control was beta-Actin (Abcam, Cat# ab8227, RRID: AB_2305186, USA) used at 0.2 μg/ml. Secondary antibodies were added for 1 h at 37°C. Secondary antibodies: anti-IgG(Goat)-HRP (Abcam, Cat# ab6885, RRID: AB_955423, USA) used at 1 μg/ml and anti-IgG(Rabbit)-HRP (BioRad, Cat# 1706515, RRID: AB_11125142, USA) used at 1 μg/ml. Chemiluminescent substrate Amersham™ ECL™ Prime and images were obtained through LI-COR^®^ Scanner. Image analysis was performed with FIJI (FIJI is just Image-J) software.

### Mouse Model

C57BL/6 male mice (7–8 weeks old) were bred in the Animal Care facility of the Facultad de Ciencias Veterinarias, Universidad Nacional de La Plata. Mice were housed under specific pathogen free conditions and allowed access to autoclaved food and water ad libitum. We used different types of stimuli: synthetic peptide p31-43, (LGQQQPFPPQQPY), sequence derived from a-gliadin protein from wheat (Genecust, Luxenburgh) and Poly I:C (Sigma Aldrich, Cat # P1530-100MG, USA). For intragastric administration, the stimulus was diluted in phosphate saline buffer (PBS), the peptide p31-43 was used at 20 μg/mice, Poly I:C was used at 30 μg/g of mice, both stimulus together and PBS as control, were given in 200 μl using a curved oral gavage needle (22G, 3.8 cm). Four or 16 h post-inoculation mice were euthanized, and intestinal samples were collected for western blot and histological analysis. All experimental procedures were performed under appropriate local ethical guidelines approved by the Institutional Animal Care and Use Committee of the Facultad de Ciencias Exactas, Universidad Nacional de La Plata (Protocol 009-27-17).

### Histological Analysis

Sections of proximal small intestine of mice were fixed in 4% w/v formalin, embedded in paraffin, and stained with H&E for histological evaluation using a Nikon Eclipse Ti fluorescence microscope with X-Cites Series 120 Q light source. Images were taken with a Nikon Digital Sight DS Ri1 camera using Nis-Elements software and measurements were performed using FIJI software. Sections were scored in a blinded fashion, with at least 30 V/C (villus height/crypt depth) ratios assessed in each mouse, while intraepithelial lymphocytes (IELs) were counted in 10 randomly well orientated villus tips and expressed as IELs/100 enterocytes.

### Gene Expression Analysis

Duodenal samples from human patients were obtained in RNA Later Solution (Biogenex) and stored until extraction at −80°C. Total RNA was isolated with RNA Spin Mini kit (GE Healthcare). Reverse transcription was performed using 1μg of the total RNA using MML-V polymerase and random primers from Molecular Probes Inc., (Invitrogen, Carlsbad, CA, USA) were used. The Real-time PCR (qPCR) was performed using an IQ-Cycler (Bio-Rad) with the SYBR Green Supermix (Invitrogen, cat 11761-100, USA). EEF1A1 transcript was used as housekeeping gene. The threshold cycle was used as indicative of relative expression level, as described by Ginzinger (28). Primer sequences used are listed in **Table 1**. The primers were purchased from a local supplier (Genbiotech, Buenos Aires, Argentina). The running protocol was the same for all the transcripts: 95°C for 10 min and 50 cycles of 60°C for 15 s, 72°C for 45 s, and 95°C for 15 s.

**Table 1:** ﻿Primers Used for RT-qPCR Analysis.

Gene	Forward primer (5´->3´)	Reverse primer (5´->3´)
*IL33*	AATCAGGTGACGGTGTTG	ACACTCCAGGATCAGTCTTG
ST2L (*IL1RL1*)	ATGTTCTGGATTGAGGCCAC	GACTACATCTTCTCCAGGTAGCAT
sST2 (*IL1RL1*)	GGCACACCGTAAGACTAAGTA	CAATTTAAGCAGCAGAGAAGCTCC
*IFNB1*	TGGGAGGCTTGAATACTGCCTCAA	TCTCATAGATGGTCAATGCGGCGT
*IFNG*	GCAGAGCCAAATTGTCTCCT	ATGCTCTTCGACCTCGAAAC
*IL10*	TCTCCGAGATGCCTTCAGCAGA	TCAGACAAGGCTTGGCAACCCA
*IRF1*	GAGGAGGTGAAAGACCAGAGCA	TAGCATCTCGGCTGGACTTCGA
*EEF1A1*	TCGGGCAAGTCCACCACTAC	CCAAGACCCAGGCATACTTGA

### Statistical Analysis

All the comparison between control subjects and active CD patients, was performed using unpaired T-tests for parametrical data or Mann-Whitney for non-parametrical data, while one-way ANOVA and Dunnett’s or Tuckey’s post-hoc test was used when more than two groups were compared. The p-value<0.05 was considered statistically significant. Statistical analysis was performed with Prism 8 software (Graph Pad, San Diego, USA).

## Results

### Active CD Patients Present Higher Levels of Circulating IL-33 and sST2

**Figure 1:**
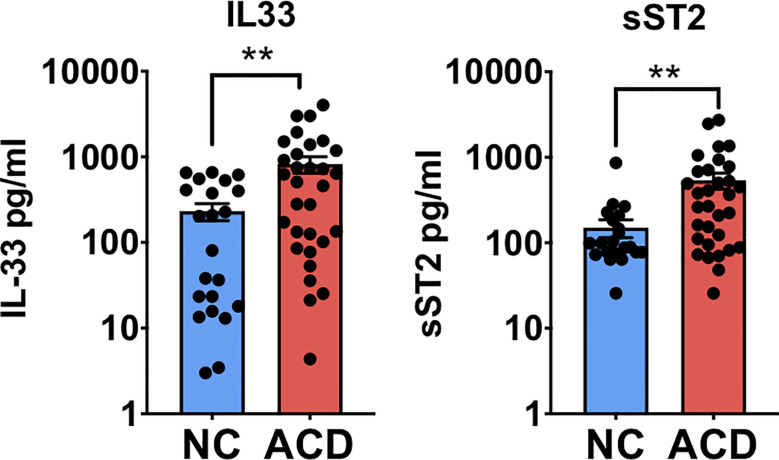
Circulating levels of IL-33 and sST2 are higher in serum of active celiac disease patients. Quantitative determination of IL-33 and sST2 was performed in serum samples of active CD (ACD) patients and non-celiac (NC) individuals using a commercial ELISA test. For IL-33, (ACD = 31, NC = 22) and for sST2, (ACD = 32, NC = 23) serum samples were analyzed. Unpaired t-test (**p < 0.01).

Since one of the most important events in IL-33 signaling is its release into the extracellular medium, we analyzed IL-33 and its decoy receptor sST2 in serum samples of a local pediatric and adult populations of active CD patients (ACD) and non-CD (NC) controls. Both IL-33 and sST2 levels were found higher in ACD patients compared with NC individuals (**Figure 1**). Pediatric and adult samples showed similar distribution and there was no difference when both populations were analyzed separately (**Supplementary Figure S1**). Moreover, there were no differences in sST2 and IL-33 serum levels when gender and enteropathy score were considered (**Supplementary Figure S1**). These findings demonstrate that a cellular source of IL-33 and sST2 is actively releasing these molecules in ACD patients.

### Duodenal Epithelium and *Lamina Propria* of Active CD Patients Show Increased Expression of IL-33 and ST2

**Figure 2:**
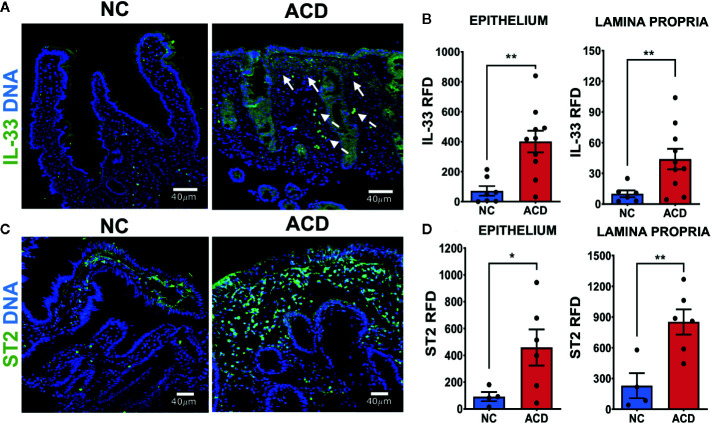
Higher expression of IL-33 and ST2 in duodenal epithelium and *lamina propria* region of active CD patients. **(A)** Representative images of IL-33 immunofluorescent staining in duodenal mucosae of NC and ACD patients. IL-33 (green), Nuclei (blue). The white arrows show some of the regions with expression of IL-33 associated to cell infiltration. Dashed arrows show cells with IL-33 nuclear localization with a morphology compatible with vascular cells. **(B)** Relative Fluorescent Density (RFD) of IL-33 in the epithelial compartment and *lamina propria*. NC (n = 7), ACD patients (n = 10). **(C)** Representative images of ST2 immunofluorescent staining in duodenal mucosae of NC and ACD patients. ST2 (green), nuclei (blue). **(D)** RFD of ST2 in the epithelial compartment and *lamina propria*. NC (n = 4) and ACD patients (n = 6). Unpaired t-test (*p < 0.05; **p < 0.01). The images in A were taken with Leica SP5 microscope with 20X objective and the images in C were taken with Zeiss Apotome with 20X objective.

To explore whether higher levels of IL-33 and sST2 found in the serum of ACD patients are linked to changes in the duodenal mucosa as a consequence of the enteropathy, indirect immunofluorescence was used to evaluate the pattern of expression of ST2 and IL-33 in duodenal mucosae sections of ACD and NC individuals. The images obtained in duodenal sections revealed that ACD patients present an increased IL-33 expression in the epithelium and crypts (**Figure 2A**). In a few cases, a similar pattern was observed in control samples but with lower fluorescence intensity (**Figure 2A**). In duodenal samples of ACD patients, areas with cell infiltration and overexpression of IL-33, close to the IL-33^+^ epithelial cells, were frequently observed (arrows in **Figure 2A**). IL-33^+^ cells scattered in the *lamina propria*, were also observed. Furthermore, both ACD patients and controls showed a strong nuclear localization of IL-33 in epithelial and *lamina propria* cells.

In order to assess the level of IL-33 expression in a semiquantitative manner, the Relative Fluorescence Density (RFD) was determined. Epithelium and *lamina propria* of ACD patients presented an increased RFD values for IL-33 labelling which were statistically higher than in control tissues (**Figure 2B**). Noteworthy, cells containing IL-33 in the nuclei were not simply scattered in the tissue, but they were distributed in a pattern resembling structures associated with microvasculature (dashed arrows, **Figure 2A**).

To understand the role of IL-33 locally, the expression of its receptor was evaluated by immunofluorescence on duodenal sections. ST2 showed a high expression in mononuclear cells of *lamina propria* and weak staining in the epithelium (**Figure 2C**). Moreover, the epithelium and *lamina propria* of ACD patients showed a statistically higher expression of ST2 compared with NC controls by semiquantitative RFD evaluation (**Figure 2D**).

### Cleaved IL-33 and sST2 Are Increased in Duodenal Tissue of Active CD Patients

**Figure 3:**
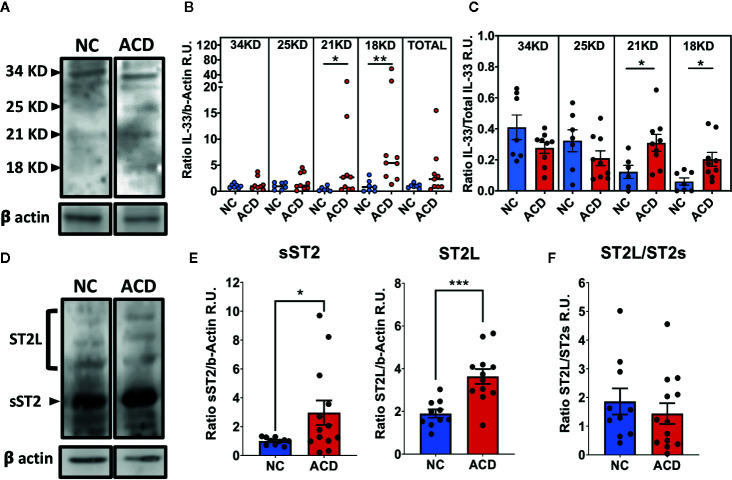
Increased levels of cleaved IL-33 fragments, ST2L and sST2 in duodenal samples of active CD patients and controls. **(A, D)** Representative images of Western blot analysis for ST2L and sST2 or the different cleaved IL-33 (25kDa, 21/20kKDa or 18kDa) or full protein (34kDa) fragments. Beta actin was used as the loading control protein in all cases. **(B)** Ratio of signal density of each of the IL-33 forms and beta-actin (NC = 7 and ACD = 9). **(C)** Relative proportion of each of IL-33 fragments for ACD and NC populations (NC = 7 and ACD = 9). **(E)** Ratio between signal density of bands corresponding to ST2s (NC = 10 and ACD = 13) or ST2L (NC = 10 and ACD = 12) and beta-actin signal. **(F)** Ratio between signal density of ST2L and ST2s for ACD and controls (NC = 10 and ACD = 12). In B, the median value is represented with a horizontal line for each population. for ACD and controls. The **C, E, F** plots are shown with average bars plus SEM bars. t-test with Welch was performed. Mann Whitney Test was performed for IL-33 relative to beta actin analysis (*p < 0.05; **p < 0.01; ***p < 0.001). Beta-Actin was used as loading control. R.U., Relative units.

Next, western blot analysis was performed to further evaluate the level of ST2 and IL-33 on whole duodenal biopsies of ACD patients and NC population. Western blot analysis showed different fragments of IL-33: a full-length band (~34 kDa), and other three protein bands with smaller molecular sizes (18, 21 and 25 kDa). The ~25 kDa band corresponds to cleaved IL-33, probably by the action of apoptotic Caspase 7 or Caspase 3 into its inactive form as described by Lüthi (29) (**Figure 3A**). The bands with a molecular mass in the range of 18-21kDa, correspond to potentially active IL-33 (3, 4). Interestingly, 18 and 21 kDa IL-33 fragments were increased in samples from ACD patients (**Figure 3B**) and this was also observed when the ratio of 18 and 21 kDa fragments were referred to total IL-33 (**Figure 3C**). These results highlight an increased bioactive cleaved IL-33 forms in duodenal tissue of ACD patients.

In addition, Western blot analysis showed that ST2L and sST2 forms were increased in duodenal tissues of ACD patients (**Figures 3D, E**). Several bands were found for ST2L that may be produced by heterogeneous glycosylation of this receptor as described by Tago (30) and Yoshida (31) while for sST2 only a band of about 57 kDa was found. Together, these results showed an increase in the total expression of ST2 on duodenal mucosae of ACD patients. Both ST2 forms were higher in ACD samples but relative levels of ST2L/sST2 were similar when ACD and NC populations were compared (**Figure 3F**), indicating a similar upregulation of both proteins in ACD patients.

### Characterization of IL-33^+^ Cell Populations in the Small Intestinal Mucosa

**Figure 4:**
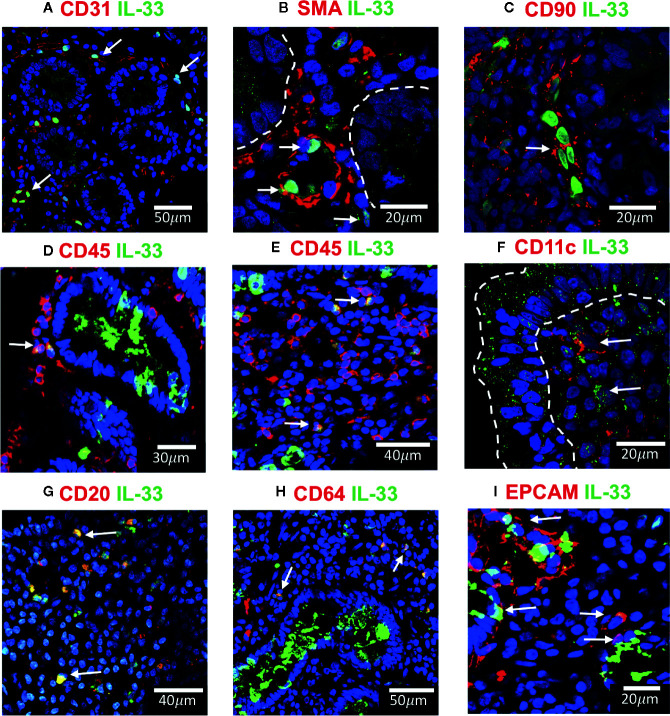
IL-33^+^ cells in Duodenal Mucosae of active celiac patients. Representative images of immunofluorescence analysis of duodenal sections of active CD patients. IL-33 (green), nuclei (blue). Other markers in red. In some images the epithelial compartment was delimited with white dashed lines. **(A).** CD31^+^ endothelial cells expressing IL-33 (white arrows). **(B)** Smooth muscle actin in red and white lines delineated intestinal crypts. The white arrows point to SMA^+^IL-33^+^ cells which are located around vascular structures and crypts. **(C)** Some of the IL-33^+^ CD90^+^ cells are pointed by a white arrow. **(D, E)** CD45+IL-33+ cells in *lamina propria*, but not in epithelial compartment. **(F)** A few IL-33^+^CD11c^+^ where close to the epithelium of ACD patients. **(G)** Some of the CD20^+^IL-33^+^ cells are pointed with white arrows. **(H)** Some of the *lamina propria* double positive IL-33^+^ CD64^+^ are pointed with white arrows. **(I)** EPCAM^+^ IL-33^+^ cells are pointed with white arrows. The images were taken with 20X and 63X objectives from the SP5 Leica Microscope and objectives 20X, 40X and 60X from the Apotome Zeiss microscope.

Since IL-33^+^ cells were found abundantly distributed in the nuclei of *lamina propria* cells with a distribution and arrangement resembling myofibroblast or endothelial cells from microvasculature-like structures (**Figure 2A**), we performed a series of double indirect immunofluorescence assays to co-stain IL-33 and characteristic markers of mesenchymal cells (SMA, CD90), dendritic cells (CD11c), macrophages/neutrophils (CD64), B cells (CD20) and the pan-leukocyte marker (CD45) (**Figure 4**).

Cells associated with structures resembling microvasculature and containing IL-33 in the nuclei were identified as endothelial CD31^+^ cells (**Figure 4A**). Other IL-33 nuclear positive cells expressed SMA and CD90 (**Figures 4B, C**) identifying them as mesenchymal cells, mainly myofibroblast and pericytes around vascular and crypt structures immersed in the *lamina propria*. Interestingly, a vast majority of IL-33^+^ cells in ACD patients were close to the villi’s epithelium (white arrows in **Figure 2A**), some of these cells presented a cytoplasmatic expression (**Supplementary Figure S2**). Moreover, using a panel of antibodies, some of these IL-33^+^ cells were characterized as CD45^+^ (**Figures 4D, E**) CD11c^+^ (**Figure 4F**), CD20^+^ (**Figure 4G**) or CD64^+^ cells (**Figure 4H**) and some were weakly CD31 positive (**Supplementary Figure S2C**). IL-33^+^ cells in the epithelial compartment where EPCAM^+^ CD45^-^ cells which identifies them as epithelial cells (**Figures 4D, I**). These findings suggest that IL-33 can be released by different types of cells from the duodenal mucosa, particularly by infiltrating mononuclear cells, like B cells, macrophages and dendritic cells. Endothelial and epithelial cells can also act as a source of IL-33 in the extracellular space upon activation.

### Analysis of *In Vitro* and *In Vivo* Expression of IL-33 Upon Proinflammatory Stimulation

**Figure 5:**
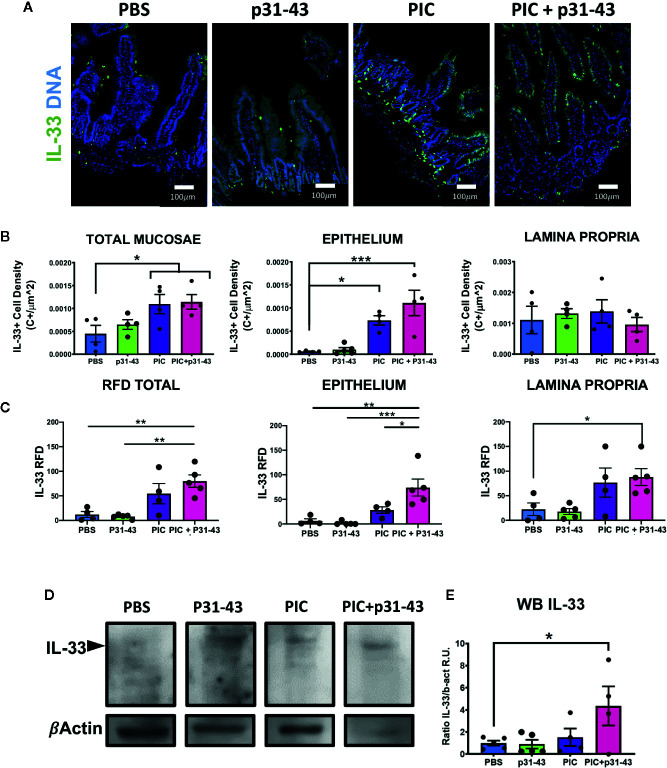
Induction of IL-33 in an acute inflammatory response in a mice model. **(A)** Representative images of immunofluorescence analysis of duodenal sections of each of mice treated with: PBS, p31-43, PIC or PIC+p31-43. The IL-33 staining is shown in green and Cell Nuclei in blue. **(B)** Results of IL-33+ cell density on total mucosae, epithelium or *lamina propria* for each of the conditions. **(C)** Results of IL-33+ RFD analysis on total mucosae, epithelium or *lamina propria* for each of the conditions. **(D)** Representative western blot analysis of IL-33 analysis in whole mucosae of each of the conditions. **(E)** Results of the analysis of IL-33 western blot for each of the conditions relative to beta actin signal and control stimuli (PBS). One-way ANOVA, post-test Tukey. (*0.01 < p < 0.05, **0.001 < p < 0.01, ***p < 0.001).

Since expression of IL-33 was up-regulated in duodenal mucosa of ACD patients, a tissue suffering from a chronic inflammatory condition, we aimed to assess whether proinflammatory stimuli can modulate IL-33 expression in this condition. Based on their relevance in CD pathogenesis, IFN*γ* (32, 33) and the gliadin peptide p31-43 (34, 35), as well as a synthetic TLR3 ligand, poly I:C, which is known to induce IL-33 expression in different cell lines and murine models (36–38) were evaluated in an *in vitro* assay using the human intestinal epithelial cell line, HT29. Poly I:C strongly induced IL-33 transcript in HT-29 cells, with no differences for the other inflammatory treatments (**Supplementary Figure S3**). According to these findings and considering previous works on an experimental model of enteropathy developed in wild type mice (34, 35), we aimed to study whether an acute inflammatory stimulus, intragastric p31-43 and poly I:C treatments, was able to upregulate IL-33 expression *in vivo*. We showed previously that intragastric treatment with p31-43, causes inflammasome activation and IL-1β production, increased number of IELs and reduction of V/C ratio (34), while poly I:C, induces an inflammatory reaction including increased number of IELs, reduction of V/C ratio, and upregulation of IFNβ and IFN*γ* expression (35). All stimuli tested were able to induce an increase of the IELs numbers and reduction of V/C ratio, confirming the inflammatory response produced upon these treatments (**Supplementary Figure S4**). IL-33 production was induced in total mucosae and particularly in epithelial cells of mice treated with poly I:C or poly I:C+p31-43, being the higher fluorescence signal found in epithelial and *lamina propria* cells from poly I:C+p31-43 treated mice (**Figures 5A–C**). Concordantly, western blot analysis showed that total IL-33 was induced predominantly in poly I:C+ p31-43 treatment (**Figures 5D, E**). This difference may be explained by a possible synergic action, since poly I:C and p31-43 may drive different signaling pathways exacerbating the inflammatory reaction on mucosal intestinal cells, mainly on epithelial cells (**Figure 5B**). This hypothesis may explain why there was no change in the number of IL-33^+^ cells in the *lamina propria* or epithelium, when poly I:C and poly I:C + p31-43 were compared, although the rate of production of IL-33 was increased. These findings suggest that IL-33 expression can be induced by signals derived upon triggering TLR3/RIGI and enhanced by proinflammatory effects of p31-43.

### ST2^+^ CD8^+^ T Lymphocytes Are Increased in CD Samples

As total ST2 expression was increased on whole tissue on both epithelial and *lamina propria* compartments of duodenum in ACD patients, we aimed to identify the ST2^+^ cells populations.

**Figure 6:**
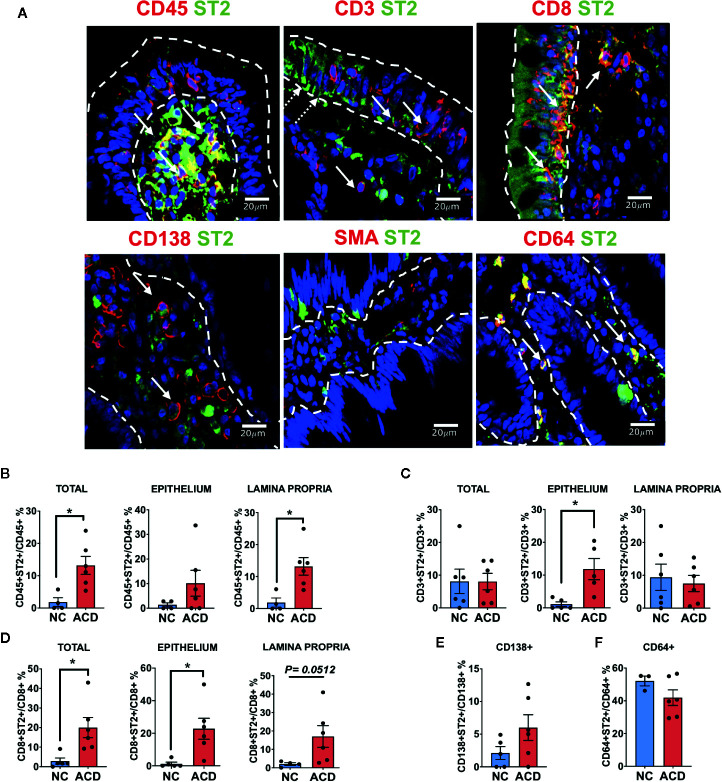
Characterization of ST2^+^ cells in duodenal mucosa. **(A)** Representative images of immunofluorescence analysis of duodenal sections of active CD patients. ST2 (green), nuclei (blue). Other markers in red. Epithelial compartment was delimited with white dashed lines. White Arrows indicate some of the double positive cells for ST2 and CD45, CD3, CD8, CD138, SMA and CD64. White dashed arrows indicate ST2 expression in epithelial cells. Counting of double positive CD45^+^ST2^+^ (NC = 4, ACD = 6). **(B)** CD3^+^ST2^+^ (NC = 5, ACD = 5). **(C)** CD8^+^ST2^+^ (NC = 5, ACD = 6). **(D)** cells in the whole mucosae (Total), in the epithelium and in the lamina propria compartment for active CD and control patients. Counting of double positive CD138^+^ST2^+^ (NC = 5, ACD = 6) **(E)** and CD64^+^ST2^+^ cells (NC = 3, ACD = 6) **(F)** in the whole duodenal mucosa. T-test was used as Statistical analysis with Welch correction if needed. The p-value < 0.05 was considered statistically significant (*p < 0.05). All the images were taken with Zeiss Apotome with objective 40X.

Since many of the ST2^+^ cells were mononuclear cells infiltrating the epithelium and *lamina propria*, most of them were hematopoietic derived CD45^+^ cells (**Figure 6A**), as we found an increase in RDF of ST2 staining in CD45^+^ (**Supplementary S5A**), as well as, in the cellular proportion of ST2^+^ cells in the CD45^+^ population in ACD patients (**Figure 6B**). We also found that many of the CD45^+^ cells were T lymphocytes (CD3^+^ cells) (**Figure 6A**). Moreover, many of the ST2^+^ cells were CD8^+^ cells scattered though the *lamina propria* and in the epithelial compartment (**Figure 6A**).

Though percentage of CD3^+^ST2^+^ in ACD and controls were similar, the amount of CD3^+^ST2^+^ cells in the epithelial compartment was significantly increased in the ACD population (**Figure 6C**). Remarkably, among T lymphocytes, we found a higher number of CD8^+^ST2^+^ cells in the epithelial compartment and in the *lamina propria* of ACD samples (**Figure 6D**). These results point out to a selective increase in the number of ST2^+^CD8^+^ T lymphocytes in the ACD population. The CD8^+^T cells also have an increase in RDF on both *lamina propria* and epithelium compartments (**Supplementary S5B**). Interestingly, we found a population of ST2^+^ cells expressing the transcription factor T-bet (**Figure S6**). These ST2^+^T-bet^+^ cells were found spread through the *lamina propria* but also in the epithelial compartment in ACD patients.

ST2^+^ cells in the epithelial compartment were identified as CD45^-^ST2^+^ or CD3^-^ST2^+^ cells and were mostly present on the luminal epithelium. Noteworthy, a high number of plasma cells (CD138^+^) with a mild cytoplasmic expression of ST2 on both ACD and controls was found. In addition, 40 to 50% of the CD64^+^ cells were ST2^+^ cells, likely macrophages (**Figures 6E, F**); however, CD64^+^ neutrophils cannot be ruled out. On the other hand, we found a few SMA^+^ cells, probably myofibroblast close to the luminal epithelium, though due to uneven SMA staining inside the cytoplasm, the counting could not be performed.

### IL-33/ST2 Axis Components Correlates Positively With IRF1 in Inflamed ACD and Healthy Duodenal Mucosa Tissue

**Figure 7:**
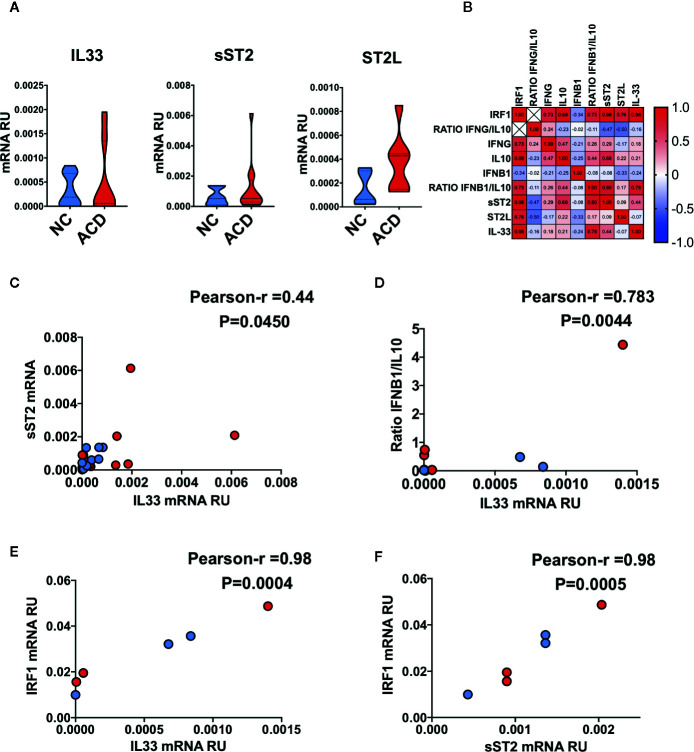
Correlation between the IL33/ST2 axis and inflammatory markers in duodenal mucosae. **(A)** Violin plots of qPCR results for IL33 gene (NC = 11, ACD = 15) and IL1RL1 splice variants corresponding to sST2 (NC = 10, ACD = 12) and ST2L (NC = 5, ACD = 7) proteins. **(B)** Correlation heatmap of Pearson r values between different genes evaluated in ACD and NC individuals. **(C)** Correlation plot of sST2 mRNA with IL33 mRNA. **(D)** Correlation plot of ratio IFNB1/IL10 with IL33 mRNA. **(E)** Correlation plot of IRF1 mRNA with IL33 mRNA. **(F)** Correlation plot of IRF1 mRNA with sST2 mRNA. All ACD samples are shown as red dots and NC as blue dots, p-value is shown for each correlation. Unpaired T-test was used as the statistical analysis in **(A)** and Pearson correlation analysis for multiple correlations of different genes in **(B–E)**. The p-value < 0.05 was considered statistically significant.

To explore the relationship between IL-33/ST2 axis with genes related with inflammatory conditions we performed a correlation analysis from ACD and healthy NC. We found that none of the IL33/ST2 axis components were upregulated in ACD patients (**Figure 7A**). Next, we assessed the potential connection of the IL-33/ST2 axis with other celiac disease associated cytokines and intracellular factors (**Figure 7B**), particularly, interferon gamma IFNG (32, 33), Interferon beta IFNB1 (39, 40), IL10 (41) and the IRF1 factor (42, 43). IFNG/IL10 and IFNB1/IL10 ratios were used as indirect indicators of proinflammatory responses in ACD population. We found that IL33 and its decoy receptor, sST2, have a mild positive correlation with each other (**Figure 7C**). Particularly, IL33 showed a mild correlation with the IFNB1/IL10 ratio (**Figure 7D**). Though none of the transcripts correlated with IFNG or IFNB1, they were linked to IFN induced transcriptional factor-1, IRF1 (**Figure 7E**). Altogether, these findings suggest a regulation of IL33 and sST2 expression by IFNs (**Figure 7F**).

## Discussion

IL-33 is a potent alarmin that can be released from different cells under stress conditions and necrotic death (44). Mouse models (9, 11, 45, 46) and disease conditions where IL-33 is released, have shown that biological consequences over the immune response depend on the tissue environment (13, 22, 23, 47). Different human disorders involve IL-33 as a potent trigger of inflammatory processes, such as Rheumatoid Arthritis, Systemic Sclerosis, Sjörgen Disease and Psoriasis (47–50). In addition, free IL-33 may act as a potent inducer of regulatory T cell (Tregs) responses (11). Alternatively, IL-33 is able to act on pro-tolerogenic dendritic cells that potentiate Tregs polarization by increasing IL-2 production. Also, IL-33 acts directly on Treg ST2L^+^ cells inducing their proliferation.

Particularly, IL-33 plays a role in gut homeostasis by regulating local microbiota through the induction of IgA (18)and antimicrobial peptide production (19). IL-33 is involved in gastrointestinal pathologies, such as ulcerative colitis, where IL-33 is upregulated and believed to potentiate Th2 responses (21, 22, 51). This is also supported by the amelioration of ulcerative colitis animal models where IL-33 signaling was blocked (52). Collectively, this not only emphasizes the crucial and dual role of IL-33 potentiating proinflammatory responses, but also promoting Treg cells and amphiregulin release from ILC2 cells (15).

Nevertheless, it is known that certain proinflammatory cytokines, such as IL-23p19 can deactivate this pro-tolerogenic mechanism of IL-33 by targeting Treg directly (11). Moreover, IFN*γ* derived from viral or bacterial infections acts as an inhibitor of IL-33 driven pro-Th2 responses, as inhibiting ILC2 responses to IL-33 (53).

The inflammatory response enables IL-33 to control cells involved in Type 2 immune responses to strongly potentiate cytotoxic activity. In a pro-Th1 environment, as reported in some viral infections, cytotoxic CD8^+^ T cells and NK cells may produce ST2L (9, 54–56) and IFNγ to expand the Th1 response (57).

In damaged tissues, release of sST2, as decoy factor, limits the effect of free IL-33 (22, 58–60) and increases IL-33 response threshold in the microenvironment where both coexist (13, 61). As mentioned above, necrotic or stress cells are believed to be a source of free IL-33. Thus, in an inflammatory setting, the relative abundance and localization of ST2^+^ cells, local sST2 release, and presence of cytokines, such as IFNγ and IL-23, will determine the biological effects of IL-33.

Celiac disease (CD), a chronic immune mediated pathology, is an example of these two functional roles of IL-33, where an exacerbated cytotoxic response against stressed cells is accompanied by an intense but ineffective pro-regulatory response (62–67).

To investigate the link between IL-33 and CD, we assessed the levels of IL-33 and ST2 in a local pediatric and adult population of CD patients at diagnosis and non-celiac controls. We found that concentration of IL-33 and sST2 in serum were higher in ACD than in NC population. Levels of both proteins were higher than those reported for a Spanish population by Lopez Casado (26), probably due to difference in the commercial test used.

Immunofluorescence assays on sections of duodenal biopsies showed that expression of IL-33 and ST2 is increased in ACD patients. Quantitative analysis of intensity of fluorescence (RFD) and western blot analysis showed that expression of IL-33 and its receptor were upregulated in duodenal mucosa in ACD patients. In contrast, the mRNA levels of IL-33 and ST2L or sST2 did not show an upregulation in ACD samples, this is probably due to a dilution effect when mRNA levels were determined in the whole tissue or difference in post-transcriptional regulation increasing IL-33 or ST2 protein expression (68–72). Overall, these findings suggest that the main source of circulating IL-33 and sST2 in ACD patients is the small intestinal mucosa. Moreover, immunofluorescence analysis confirmed that IL-33 is produced by a great variety of cells. Particularly, IL-33 was found in epithelial cells, endothelial cells and myofibroblasts. In fact, in many of these cells, IL-33 was found having a nuclear and cytoplasmic localization, suggesting its potential release from these cells (51, 73, 74). Furthermore, IL-33^+^ cells were also found in the inflammatory infiltrate, corresponding to CD11c^+^ dendritic cells, CD20^+^ B cells, and CD64^+^ cells, probably monocytes/macrophages. Accordingly, upon stimulation with gliadin peptides, PBMCs from ACD patients were able to release IL-33 in the culture medium (26).

The release of IL-33 may induce different immune responses depending on the target cells present in the surrounding tissue. In intestinal mucosa in CD enteropathy, NK, NKT and cytotoxic T cells are potential targets for IL-33 (26). These cell populations, increased in CD duodenum, are known to play an important role in CD pathology (75, 76), and are also targeted by free IL-33 on viral infections (46) and certain tumor environments (77). Significantly, IL-33 can enhance cytotoxic responses and induce IFN*γ* release by T CD8^+^ cells, by potentiating the effects of IL-12 and enhancing T-bet and BLIMP1 expression (9, 13, 54, 78). In this scenario, IL-33 could enhance cytotoxic mechanisms and pro-Th1 immune response in CD pathogenesis. The increased number of CD8^+^ST2^+^ cells on both epithelial and *lamina propria* compartments pointed toward this hypothesis. However, we cannot rule out the possibility of IL-33 acting on Treg, ILC2 or Macrophage/Dendritic cells to promote different immune responses.

Levels of sST2 were higher in peripheral blood and duodenal tissue of ACD patients, suggesting that it may act as a decoy factor in intestinal tissue modulating the biological activity of IL-33. However, upregulation of both splice variants of ST2: ST2L and sST2 and sST2/ST2L ratio were similar in ACD and NC populations, suggesting that target ST2^+^ cells may respond to free IL-33 in ACD intestinal mucosa when its local concentration exceeds the sST2 levels.

Since IL-33 may have different functions according with the tissue environment (61), distinct scenarios can be proposed in small intestine in ACD. Through Tregs and ILC2 cells, IL-33 would participate as a pro-tolerogenic and wound healing cytokine, but these effects can be inhibited by sST2. Accordingly, higher levels of sST2, as a proinflammatory factor, were associated with tissue damage and inhibition of the wound healing process induced by IL-33 (22, 58, 79). Additionally, IFNγ and IL-23, cytokines produced upon gluten stimulation in ACD (33, 80), are strong inhibitors of IL-33 induced effects on ILC2, Th2 and Treg cells (11, 53). Since we found an increase in CD8^+^ST2^+^ cells in small intestine in ACD, a disorder characterized by a Th1 response and cytotoxic activity carried out by CD8^+^ T cells (67, 75, 81), the scenario where IL-33 potentiates cytotoxic functions is more likely to happen.

Posttranslational modifications of IL-33 are critical for its interaction with ST2L. These modifications depend on the activity of specific proteolytic enzymes, which may be induced or activated under specific conditions (13). Particularly, proteases as effector caspases in apoptosis and some proinflammatory enzymes secreted by Neutrophils and Mast cells can substantially modified IL-33 bioactivity. The cleavage of IL-33 by caspase-3 or 7 during apoptosis renders inactive IL-33 fragments of about 25 kDa and 6.5 kDa (29). Our western blot setup cannot detect fragments under 12 kDa threshold. Therefore we did detect the 25 kDa fragment but the 6.5 KDa fragments could not be analyzed. Interestingly, ACD patients present active apoptotic mechanisms in different cells, but particularly in epithelial cells (82, 83). As a consequence, IL-33^+^ cells such as epithelial or endothelial cells, may undergo apoptosis in CD intestinal mucosae and produce inactive 25 kDa IL-33 fragments. Unlike apoptosis, other programmed cell death mechanisms such as pyroptosis and necroptosis release cellular components and alarmins as IL-33 with proinflammatory properties to the extracellular compartment (84–86). However, we cannot assumed that all the 25 kDa fragments are derived from apoptotic cells, since Tryptase from Mast cells cleaves full IL-33 yielding fragments between the aminoacidic positions 72–270 and 79–270 which have a mass of about 25 kDa (4). Additionally, we detected 18 and 20/21 kDa IL-33 fragments having stronger bioactive effect than native full-length IL-33 (3, 4). These IL-33 fragments result from digestion by enzymes, such as cathepsin G, neutrophil elastase, tryptase or chymase, derived from neutrophil or mast cells. Lefracais et al, (3, 4) have reported that enzymes secreted by Neutrophils (Cathepsin G and Neutrophil Elastase) or Mast cells (Chymase, Tryptase) can produce fragments of about 18/19 and 20/21 KD comprising aminoacidic positions: 95–270, 99–270, 107–270, and 109–270 from full IL-33. Since our western blot sensitivity did not allow us to differentiate variation of about 1 kDa in the fragments mass, we considered these 18/21 kDa fragments as mature forms of IL-33 reported in literature (3, 4). Interestingly, both mast cells (87, 88) and neutrophils (89) are increased in number and activity in duodenal mucosa in active CD, suggesting both increased IL-33 release and its processing in the extracellular space occurs simultaneously, resulting in inflammatory duodenal mucosa environment in ACD patients. Moreover, based on their proinflammatory activity, 20/21 kDa IL-33 fragments have also been proposed as a potent vaccine adjuvant to enhance IFN*γ* ^+^ CD8^+^ T cell responses against certain viruses, such as HPV (90, 91). As shown here, some of the CD8^+^ST2^+^ cells were found in the intraepithelial compartment, where intraepithelial lymphocytes, recognized as tissue-resident cytotoxic T lymphocytes playing a critical role in CD pathogenesis (75, 92). Interestingly, the transcription factor T-bet, master transcription factor for differentiation of Th1 and cytotoxic T lymphocytes (93), was found in a population of ST2^+^ cells spread through the *lamina propria* and in the epithelial compartment in CD patients. Collectively, we suggest that recently released IL-33 can be processed by enzymes from mast cells or neutrophils, resulting in 18/21 kDa fragments with increased affinity to ST2L and higher potency to activate cytotoxic ST2L^+^CD8^+^ T cells in duodenal mucosae of CD patients.

When IL33/ST2 axis transcript were evaluated, a mild correlation was found. This is concordant with reports showing that IL-33 controls the transcript levels of ST2L and sST2 in a concentration dependent manner (94). Interestingly, IL-33 and sST2 transcript correlated with IRF-1 mRNA levels, which was found to be essential for IL-33 production under viral infections in endothelial cells (95). Type I and II IFNs regulate IRF-1 expression in different cells (43, 96, 97), suggesting a possible indirect regulation of IL-33 expression by IFNs highlighting the need to further investigate the link between IL-33/ST2 axis and IFNs in the context of CD.

## Conclusions

The inflammatory network operating in small intestine mucosa is a consequence of an immune response to wheat proteins in CD patients. On a normal diet, local inflammation can be exacerbated through IL-33 release by programmed cell death and further production of 18/21 kDa IL-33 fragments, additionally potentiating cytotoxic ST2L^+^ CD8^+^ T cells. Since these mechanisms can be potentiated in tissues where IL-33 is secreted or released by means of proinflammatory death mechanisms, different IL-33 target cell populations may play a role in the pathogenesis of celiac disease.

## Data Availability Statement

Requests to access the datasets should be directed to fchirdo@biol.unlp.edu.ar.

## Ethics Statement

The studies involving human participants were reviewed and approved by: Ethical Committees of both Public Health Institutions. Hospital San Martin (La Plata). Hospital Sor María Ludovica (La Plata) approved this study. Written informed consent to participate in this study was provided by the participants’ legal guardian/next of kin. The animal study was reviewed and approved by the Institutional Animal Care and Use Committee of the Exact Sciences Faculty, National University of La Plata (Protocol 009-27-17).

## Author Contributions

FP acquired data, analyzed and interpreted data, and wrote the manuscript. CR and FP collected and processed the human samples. CR, EM, and PC performed the experiments in the animal model. KD-C performed the ELISA tests and co-wrote the manuscript. LaG and LuG performed the clinical evaluation and endoscopy procedures. MH contributed to data interpretation and to co-write the manuscript. FC designed the research, analyzed data and co-wrote the manuscript. All authors contributed to the article and approved the submitted version.

## Funding

This work was supported by the grants PICT 2015 1246, and PICT 2017 0880 from the Agencia Nacional de Promoción Científica y Tecnológica from Ministerio de Ciencia, Tecnología e Innovación Productiva, República Argentina. The funders had no role in study design, data collection and analysis, decision to publish or preparation of the manuscript.

## Conflict of Interest

The authors declare that the research was conducted in the absence of any commercial or financial relationships that could be construed as a potential conflict of interest.
