# Supplementary material for: IL-33 Alarmin and Its Active Proinflammatory Fragments Are Released in Small Intestine in Celiac Disease
Source: Front Immunol. 2020 Oct 8;11:581445. doi: 10.3389/fimmu.2020.581445 (PMC7578377; doi:10.3389/fimmu.2020.581445)
Supplement: Supplementary file 1 [file DataSheet_1.pdf]

Patient ID	Gender	Age (years)	Age Cohort	Clinical Diagnose	Oberhüber Score	Positivity of Serologic CD Ab	IgA Deficiency	Familiar with autoinmune disease	Familiar with Celiac Disease(CD)	Presence of other autoinmune disease	ELISA	IFIs
A#040	F	29	Adult	NC	Normal	No	No	No	No	No	Yes	No
A#041	F	83	Adult	NC	Normal	No	No	No	No	No	Yes	No
A#043	F	65	Adult	NC	Normal	No	No	No	No	No	Yes	No
A#082	F	49	Adult	NC	Normal	No	No	No	No	No	Yes	No
A#049	F	40	Adult	ACD	3	Yes	No	No	No	No	Yes	No
A#057	F	60	Adult	ACD	3	Yes	No	Yes (CD)	Yes	No	Yes	No
A#061	F	36	Adult	ACD	Normal	No	No	Yes (CD)	Yes	No	Yes	No
A#089	F	28	Adult	ACD	4	Yes	No	No	No	No	Yes	No
P#144	F	41	Adult	NC	Normal	No	No	No	No	No	Yes	No
A#121	F	47	Adult	NC	Normal	No	No	No	No	No	Yes	No
A#091	F	67	Adult	ACD	4	Yes	No	No	No	No	Yes	No
A#123	F	29	Adult	ACD	4	Yes	No	No	No	No	Yes	No
P#171	F	49	Adult	NC	Normal	No	No	No	No	No	Yes	No
P#181	F	50	Adult	NC	Normal	No	No	No	No	No	Yes	No
A#122	F	66	Adult	NC	Normal	No	No	No	No	No	Yes	No
A#117	F	36	Adult	ACD	4	Yes	No	No	No	No	Yes	No
A#126	F	60	Adult	ACD	3	Yes	No	No	No	No	Yes	No
A#129	F	22	Adult	ACD	4	Yes	No	No	No	No	Yes	No
A#124	F	24	Adult	ACD	3	Yes	No	No	No	No	Yes	No
P#187	F	56	Adult	NC	Normal	No	No	No	No	No	Yes	No
A#167	F	41	Adult	NC	Normal	No	No	No	No	No	Yes	No
A#164	F	19	Adult	ACD	3	Yes	No	Yes (CD)	Yes	No	Yes	No
A#151	F	45	Adult	ACD	4	Yes	No	No	No	No	Yes	No

P#189	F	41	Adult	NC	Normal	No	No	No	No	No	Yes	No
A#174	F	38	Adult	NC	Normal	No	No	No	No	No	Yes	No
P#020	F	60	Adult	NC	Normal	No	No	No	No	No	Yes	No
P#249	F	42	Adult	NC	Normal	No	No	Yes (CD)	Yes	No	Yes	No
P#192	F	16	Adult	NC	Normal	No	No	No	No	No	Yes	No
A#202	F	60	Adult	ACD	1	Yes	No	No	No	No	Yes	No
A#155	F	27	Adult	ACD	4	Yes	No	No	No	No	Yes	No
A#156	F	50	Adult	ACD	4	Yes	No	No	No	No	Yes	No
P#193	F	40	Adult	NC	Normal	No	No	No	No	No	Yes	No
P#194	F	19	Adult	NC	Normal	No	No	No	No	No	Yes	No
A#165	F	30	Adult	ACD	3	Yes	No	No	No	No	Yes	No
A#170	F	60	Adult	ACD	4	Yes	No	No	No	No	Yes	No
A#172	F	55	Adult	ACD	3	Yes	No	No	No	No	Yes	No
P#195	F	54	Adult	NC	Normal	No	No	No	No	No	Yes	No
P#196	F	15	Adult	NC	Normal	No	No	No	No	No	Yes	No
A#176	F	65	Adult	ACD	4	Yes	No	No	No	No	Yes	Yes
A#179	F	45	Adult	ACD	3	Yes	No	No	No	No	Yes	No
P#200	F	60	Adult	NC	Normal	No	No	No	No	No	Yes	No
P#207	F	45	Adult	NC	Normal	No	No	No	No	No	Yes	No
A#328	F	54	Adult	NC	Normal	No	No	No	No	No	No	Yes
A#332	F	47	Adult	NC	Normal	No	No	No	No	No	No	Yes
A#334	F	60	Adult	NC	Normal	No	No	No	No	No	No	Yes
A#059	M	63	Adult	ACD	4	Yes	No	No	No	No	Yes	No
A#338	F	67	Adult	NC	Normal	No	No	No	No	No	No	Yes
A#038	M	57	Adult	NC	Normal	No	No	No	No	No	Yes	No
A#259	M	16	Adult	ACD	1	Yes	No	No	No	No	Yes	No
A#263	M	16	Adult	ACD	3	Yes	No	No	No	No	Yes	No
A#083	M	51	Adult	NC	Normal	No	No	No	No	No	Yes	No

P#073	M	66	Adult	NC	Normal	No	No	No	No	No	Yes	No
P#215	M	38	Adult	NC	Normal	No	No	No	No	No	Yes	No
P#216	M	37	Adult	NC	Normal	No	No	No	No	No	Yes	No
A#337	M	45	Adult	NC	Normal	No	No	No	No	No	No	Yes
P#005	F	2	Pediatric	ACD	4	Yes	No	No	No	No	Yes	No
P#208	F	6	Pediatric	NC	Normal	No	No	No	No	No	Yes	No
P#209	F	3	Pediatric	NC	Normal	No	No	No	No	No	Yes	No
P#225	F	3	Pediatric	NC	Normal	Yes	No	No	No	No	Yes	No
A#181	F	7	Pediatric	ACD	3	Yes	No	Yes (CD)	Yes	No	Yes	No
P#069	F	5	Pediatric	ACD	4	Yes	No	No	No	No	Yes	No
A#199	F	7	Pediatric	ACD	4	Yes	No	No	No	No	Yes	Yes
A#200	F	10	Pediatric	ACD	3	Yes	No	No	No	No	Yes	No
A#238	F	11	Pediatric	ACD	Normal	No	No	No	No	No	Yes	No
P#226	F	4	Pediatric	ACD	1	Yes	No	No	No	No	Yes	No
P#210	F	5	Pediatric	NC	Normal	No	No	No	No	No	Yes	No
P#212	F	14	Pediatric	NC	Normal	No	No	No	No	No	Yes	Yes
P#013	M	5	Pediatric	NC	Normal	No	No	No	No	No	Yes	No
A#243	F	7	Pediatric	ACD	4	Yes	No	No	No	No	Yes	No
P#019	M	3	Pediatric	NC	Normal	No	No	No	No	No	Yes	No
P#217	M	5	Pediatric	NC	Normal	No	No	No	No	No	Yes	No
A#248	F	4	Pediatric	ACD	4	Yes	No	Yes (CD)	CD	No	Yes	No
A#250	F	13	Pediatric	ACD	3	Yes	No	No	No	No	Yes	No
P#146	M	3	Pediatric	NC	Normal	No	No	No	No	No	Yes	No
P#232	F	1	Pediatric	ACD	1	Yes	No	No	No	No	Yes	No
A#252	F	7	Pediatric	ACD	3	Yes	No	No	No	No	Yes	No
P#166	M	8	Pediatric	NC	Normal	No	No	No	No	No	Yes	No
A#256	F	8	Pediatric	ACD	4	Yes	No	No	No	No	Yes	Yes
P#241	F	7	Pediatric	ACD	2	Yes	No	Yes (CD)	Yes	No	No	Yes

P#242	F	5	Pediatric	ACD	2	Yes	No	No	No	No	Yes	No
P#173	M	14	Pediatric	NC	Normal	No	No	No	No	No	Yes	No
A#258	F	6	Pediatric	ACD	4	Yes	No	No	No	No	Yes	Yes
P#252	F	2	Pediatric	ACD	2	Yes	No	No	No	No	Yes	Yes
P#328	F	10	Pediatric	ACD	2	Yes	No	No	No	No	No	Yes
P#186	M	9	Pediatric	NC	Normal	No	No	No	No	No	Yes	No
P#336	F	10	Pediatric	ACD	4	Yes	No	No	No	No	No	Yes
P#339	F	5	Pediatric	ACD	4	Yes	No	No	No	No	No	Yes
P#027	M	9	Pediatric	ACD	4	Yes	No	No	No	No	Yes	No
P#061	M	9	Pediatric	ACD	4	Yes	No	No	No	No	Yes	No
P#191	M	1	Pediatric	NC	Normal	No	No	No	No	No	Yes	Yes
P#063	M	12	Pediatric	ACD	3	Yes	No	No	No	No	Yes	No
P#218	M	2	Pediatric	NC	Normal	No	No	No	No	No	Yes	No
A#267	M	10	Pediatric	ACD	2	Yes	No	No	No	No	Yes	No
P#169	M	5	Pediatric	ACD	4	Yes	No	No	No	No	Yes	No
A#270	M	3	Pediatric	ACD	3	Yes	No	No	No	No	Yes	No
P#185	M	4	Pediatric	ACD	4	Yes	No	No	No	No	Yes	No
A#271	M	6	Pediatric	ACD	1	Yes	No	No	No	No	Yes	No
P#233	M	3	Pediatric	ACD	1	Yes	No	No	No	No	Yes	No
P#222	M	5	Pediatric	NC	Normal	No	No	No	No	No	Yes	No
P#223	M	5	Pediatric	NC	Normal	No	No	No	No	No	Yes	No
A#272	M	5	Pediatric	ACD	4	Yes	No	No	No	No	Yes	Yes
A#276	M	9	Pediatric	ACD	3	Yes	No	No	No	No	Yes	Yes
A#277	M	4	Pediatric	ACD	1	Yes	No	No	No	No	Yes	No
P#234	M	2	Pediatric	ACD	1	Yes	No	No	No	No	Yes	No
P#240	M	7	Pediatric	NC	Normal	No	No	No	No	No	No	Yes
P#244	M	10	Pediatric	ACD	Normal	No	No	No	No	No	Yes	No
P#263	M	3	Pediatric	NC	Normal	No	No	No	No	No	No	Yes

P#296	M	13	Pediatric	NC	Normal	No	No	No	No	No	No	Yes
A#317	M	5	Pediatric	ACD	4	Yes	No	No	No	No	Yes	No
P#012	M	1	Pediatric	ACD	1	Yes	No	No	No	No	Yes	No
P#247	M	11	Pediatric	ACD	4	Yes	No	No	No	No	Yes	Yes
P#297	M	4	Pediatric	ACD	2	Yes	No	Yes (T1D)	No	T1D	No	Yes
P#330	M	4	Pediatric	ACD	4	Yes	No	No	No	No	No	Yes
P#337	M	14	Pediatric	ACD	3	Yes	No	No	No	No	No	Yes
P#338	M	3	Pediatric	ACD	4	Yes	No	No	No	No	No	Yes
P#340	M	5	Pediatric	NC	Normal	No	No	No	No	No	No	Yes

Supplementary Table 1: Relevant Characteristics of Patients

In Gender Column, “F” is for Female and “M” for Male subjects. Adults samples from patients older than 15 years old were obtained from Hospital San Martin of La Plata. Pediatric samples from patients younger than 15 years old were obtained from Hospital Sor María Ludovica of La Plata. The clinical enteropathy score is based on Oberhüber score. The positivity of CD antibodies shows if patients were positive to either IgA or IgG auto antibodies: anti-transglutaminase 2, anti-deamidated gliadin peptides and anti-endomysium antibodies. ELISA and IFIs columns show if patients samples were used in ELISAs of IL33 or sST2 and/or any Fluorescence analysis of mucosal biopsies. CD: Celiac Disease, T1D: Type 1 Diabetes, ACD: active CD and NC: non celiac patient.
