# Supplementary figures and images for: IL-33 Alarmin and Its Active Proinflammatory Fragments Are Released in Small Intestine in Celiac Disease
Source: Front Immunol. 2020 Oct 8;11:581445. doi: 10.3389/fimmu.2020.581445 (PMC7578377; doi:10.3389/fimmu.2020.581445)

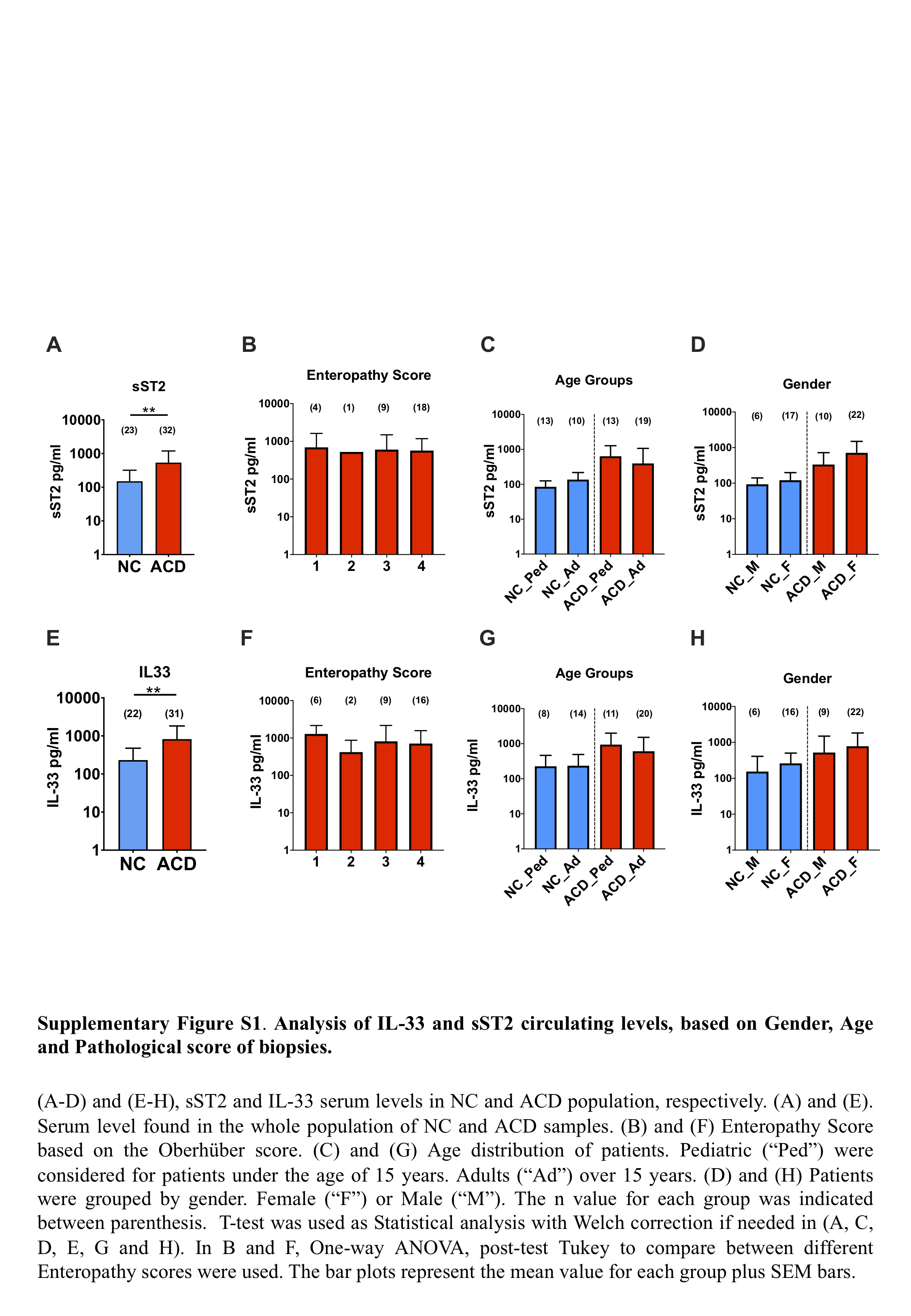

Supplement: Supplementary file 2 [file Image_1.tiff]

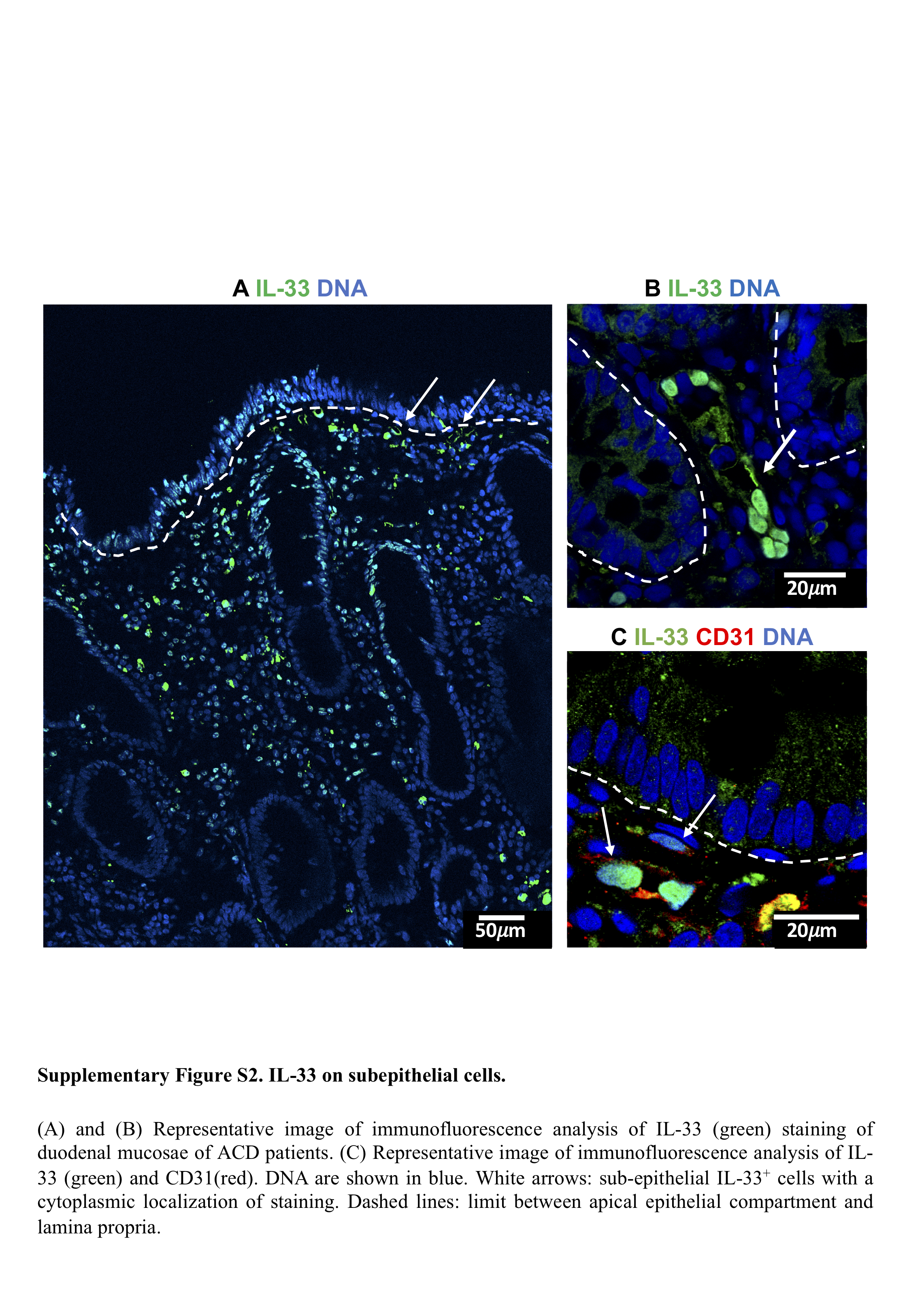

Supplement: Supplementary file 3 [file Image_2.tiff]

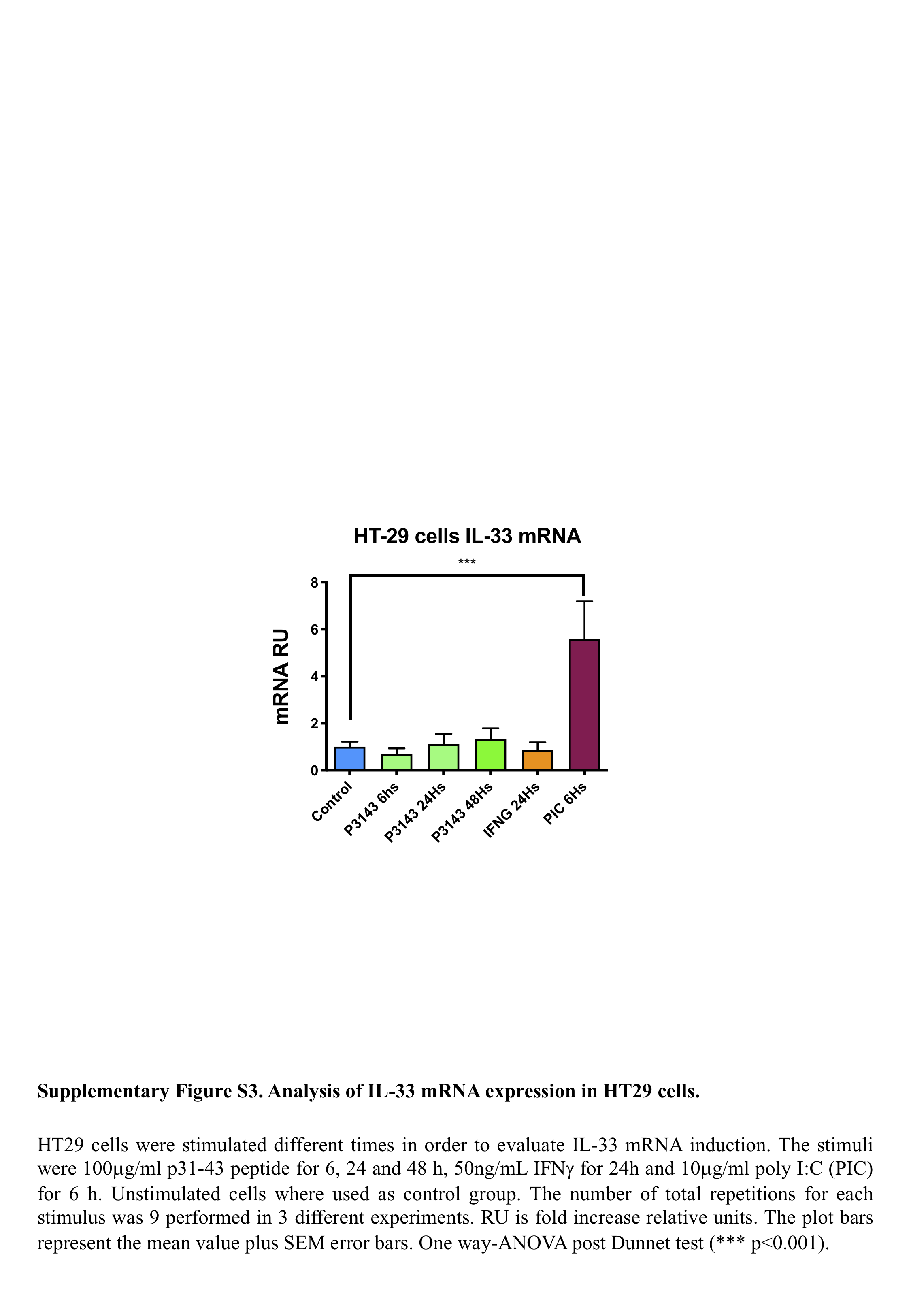

Supplement: Supplementary file 4 [file Image_3.tiff]

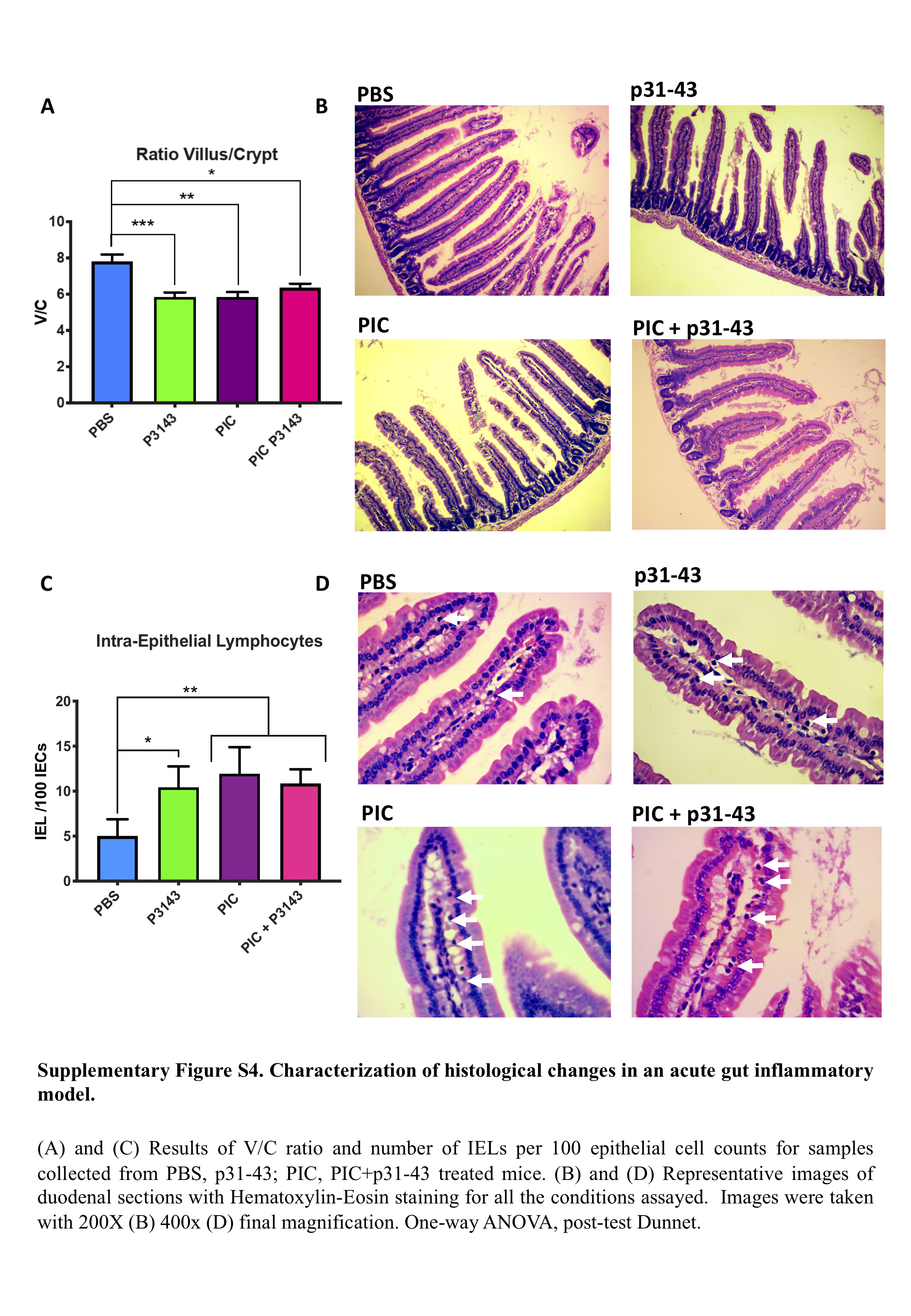

Supplement: Supplementary file 5 [file Image_4.tiff]

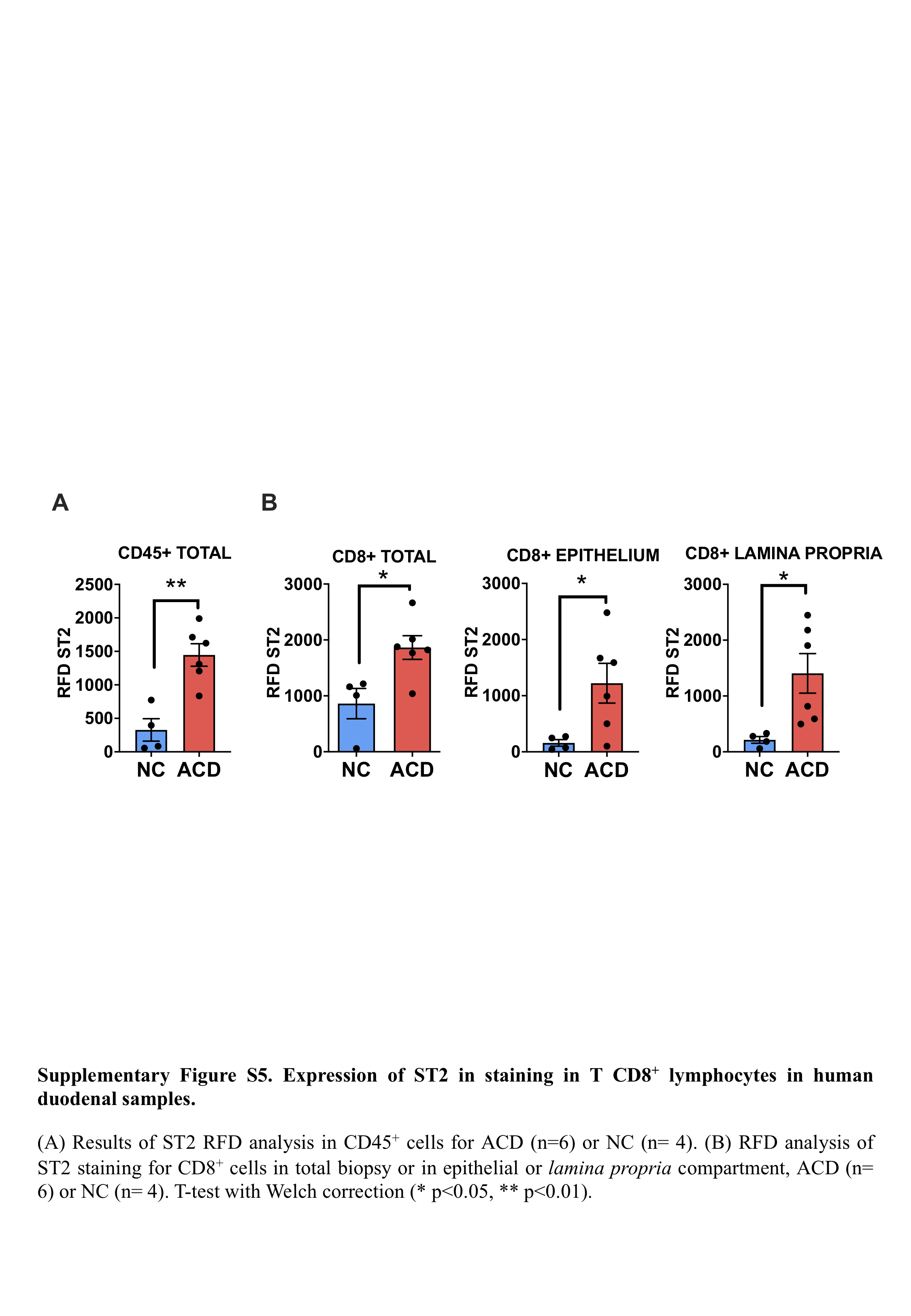

Supplement: Supplementary file 6 [file Image_5.tiff]

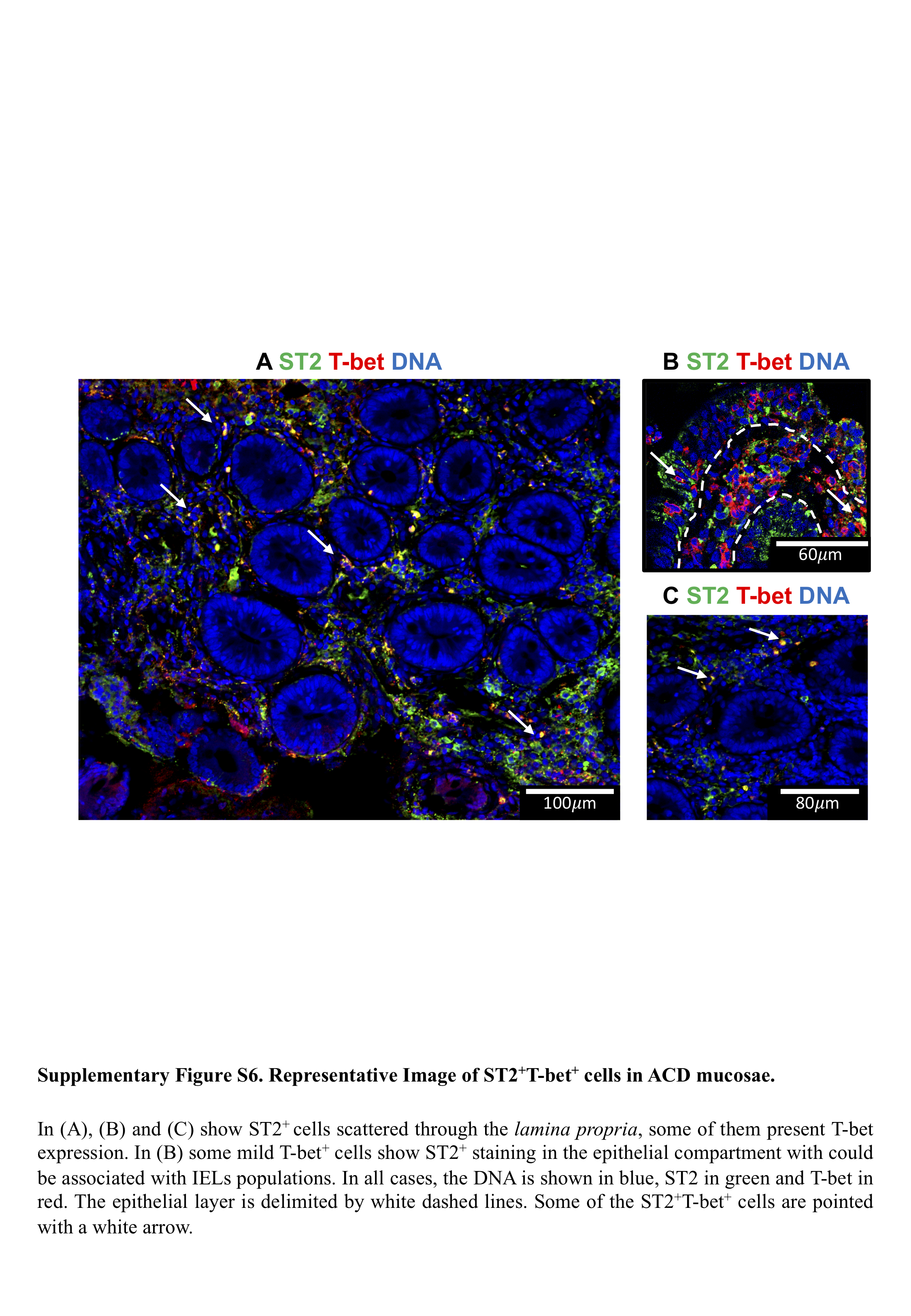

Supplement: Supplementary file 7 [file Image_6.tiff]
